# Cost-effectiveness analysis of drug-eluting beads and conventional transarterial chemoembolization in the treatment of hepatocellular carcinoma

**DOI:** 10.3389/fpubh.2022.963058

**Published:** 2022-10-26

**Authors:** Guoliang Shao, Jingwen Wang, Xiaoying Zhou, Guojun Sun, Zuojun Dong

**Affiliations:** ^1^Interventional Therapy, Cancer Hospital of the University of Chinese Academy of Sciences (Zhejiang Cancer Hospital), Hangzhou, China; ^2^Precision Innovation Center of the Diagnosis and Treatment of Hepatobiliary and Pancreatic Disease of Zhejiang University, Hangzhou, China; ^3^College of Pharmaceutical Science, Zhejiang University of Technology, Hangzhou, China

**Keywords:** DEB-TACE, cTACE, partition survival model, cost-effectiveness analysis, hepatocellular carcinoma

## Abstract

**Objective:**

To conduct a cost-effectiveness analysis of drug-eluting beads transcatheter arterial chemoembolization (DEB-TACE) and conventional transcatheter arterial chemoembolization (cTACE) for first-line treatment of hepatocellular carcinoma (HCC) from the perspective of the Chinese healthcare system.

**Methods:**

Based on the real-world clinical data of HCC patients receiving interventional therapy, a partitioned survival model was constructed for cost-effectiveness analysis. The model period is 1 month, and the research time limit is 10 years. The incremental cost-effectiveness ratio (ICER) is used as the evaluation index. One-way sensitivity analysis and probabilistic sensitivity analysis were used to analyze the uncertainty of parameters to test the stability of the model results.

**Results:**

The ICER of the DEB-TACE group was 11,875.62 $/QALYs, which was lower than the willingness to pay threshold (WTP) of 31,499.23 $/QALYs. One-way sensitivity analysis suggested that the utility value of progression-free survival (PFS) in the DEB-TACE group had the greatest impact. Probabilistic sensitivity analysis showed that at the level of WTP of 31,499.23 $/QALYs, DEB-TACE had a cost-effective probability of 92%.

**Conclusion:**

Under the current economic level in my country, DEB-TACE is more cost-effective than cTACE in the treatment of HCC patients.

## Introduction

Primary Hepatic Carcinoma (PHC) is a malignant tumor of the digestive system with a high mortality rate worldwide. Global Cancer Statistics 2020 is a statistical report on cancer worldwide, published jointly by the International Agency for Research on Cancer (IARC) and the World Health Organization (WHO). The report pointed out that in 2020, there were 19.3 million new cancer cases worldwide and 10 million deaths. Among them, primary liver cancer accounts for approximately 906,000 new cases and 830,000 deaths, making it the sixth most common malignancy and the third leading cause of death worldwide (1). In 2015, a cancer statistic about China showed that liver cancer had the fourth highest incidence rate and the second highest mortality rate (after lung cancer), with an estimated 370,000 new cases and 326,000 cancer-related deaths (2). Hepatocellular carcinoma (HCC) is the main type of PHC, accounting for about 75–85% of all cases. In Northeast Asia and Southeast Asia, including China, Indonesia and South Korea, Hepatitis B is the most important factor in causing HCC (3). In particular, as the country with the heaviest hepatitis B burden in the world, nearly half of the new cases of liver cancer patients in the world come from my country. The annual cost is 43,310.148 yuan. With the increase of laboratory fees, operation fees and inspection fees, the treatment costs of patients are increasing year by year, causing a heavy economic burden to patients (4).

Radical therapy such as surgical resection is the main treatment for early stage HCC, while hepatic artery embolization, systemic chemotherapy, and molecular targeted therapy are the main treatments for intermediate and advanced HCC (5, 6). Because the onset of HCC is relatively insidious and has no obvious early signs, it is often diagnosed at an advanced stage. At this time, the most traditional treatment methods such as surgical resection have not achieved the best results, and the prognosis is poor and the mortality rate is high (7). Transcatheter arterial chemoembolization (TACE) is currently the most widely used clinical interventional method for mid-stage HCC (8). In 2020, the Chinese Society of Clinical Oncology pointed out in the “*Guidelines for the Diagnosis and Treatment of PHC 2020”* that TACE can be used as a first-line therapy for advanced unresectable HCC. According to the different embolic agents, TACE is divided into conventional TACE (cTACE) and Drug-eluting Beads TACE (DEB-TACE) (9). cTACE is an emulsion made of lipiodol as an embolic agent, and a mixture of chemotherapy drugs and lipiodol is injected into the artery supplying the tumor. Simultaneous embolization of blood vessels, treatment of tumor necrosis through cytotoxicity and ischemia (10). However, due to the fluidity of lipiodol, the chemotherapeutic drugs cannot be accurately released around the tumor, which reduces the local effective concentration and action time of the chemotherapeutic drugs (11). DEB-TACE is a new embolization technology using drug-loaded microspheres as embolizing agent, which can accurately and permanently embolize arterial vessels and target cancer cells. It uses the ion exchange mechanism to controllably and slowly release chemotherapeutic drugs to achieve continuous drug delivery and permanent embolization, and to increase the local intratumoral drug concentration. Thus, the concentration of chemotherapeutic drugs in the systemic blood circulation is reduced, and the systemic toxicity to the human body is reduced (12).

Compared with cTACE, the drug-loaded microspheres used in DEB-TACE are expensive. In 2016, Cucchetti A et al. constructed a Markov model to compare the cost of treatment and the therapeutic effect obtained by patients after cTACE and DEB-TACE treatment, respectively (13). The results show that DEB-TACE is more cost-effective than cTACE. However, no incremental analysis of costs and effects was conducted in the study, and the results obtained have certain limitations. Currently, there is no economic evaluation of these two treatments in China. Therefore, from the perspective of the medical and health system, this paper conducts a cost-effectiveness analysis of DEB-TACE and cTACE in the treatment of HCC, and provides decision-making suggestions for the treatment of clinical HCC.

## Materials and methods

### Clinical data

A total of 89 patients with HCC who met the inclusion criteria in the interventional treatment department of the Cancer Hospital Affiliated to the University of Chinese Academy of Sciences (Zhejiang Cancer Hospital) from 2019 to 2020 was retrospectively analyzed, including 40 in the DEB-TACE group and 49 in the cTACE group. The experimental group was treated with drug-loaded microsphere embolic agent for DEB-TACE, and the control group was treated with lipiodol for cTACE.

### Inclusion and exclusion criteria

Inclusion criteria: (1) diagnosed with liver cancer by imaging and pathological examinations; (1) aged ≥18 years; (3) Barcelona Clinic Liver Cancer (BCLC) stages A to C; (4) liver function Child-Pugh grade is A or B; (5) Eastern Cooperative Oncology Group performance status (ECOG PS) score is 0–2; (6) No other disease affecting survival, survival >3 months; (7) No other treatment was performed before surgery.

Exclusion criteria: (1) Child-Pugh C grade of liver function; (2) Multiple tumor metastases throughout the body; (3) The existence of hepatic artery-portal venous fistula and hepatic artery-hepatic venous fistula.

The baseline characteristics of the patients are shown in Table 1.

**Table 1:** Baseline characteristics of the patient.

	**DEB-TACE**	**cTACE**	**P**
Patient (case)	40	49	
Gender (Male/Female)	12905	14855	0.474
Age (years)	56.0 ± 9.24	59.15 ± 9.40	0.15
**Pathological diagnosis**			
HBV	30	30	0.594
Others	10	10	
**Child-Pugh**			
A	35	47	0.266
B	4	2	
**BCLC**			
A	0	13	1
B	31	31	
C	9	6	
**ECOG PS**			
0	13	24	0.116
1	27	25	

HBV, Hepatitis B; BCLC, Barcelona Clinic Liver Cancer; ECOG PS, Eastern Cooperative Oncology Group performance status.

### Interventions

Relevant tests and examinations were performed before admission, including biochemistry, blood routine, coagulation routine, quantitative detection of hepatitis B virus DNA amplification, tumor marker screening materials, CT and MR. Interventional therapy was performed after the patient signed the informed consent to exclude the contraindication of interventional therapy.

In the DEB-TACE group, microspheres loaded with epirubicin or raltitrexed were selectively injected into the blood vessels of the tumor for embolization. When the tumor diameter was <7 cm, drug-loaded microspheres of 100–300 μm were used; when the tumor diameter was >7 cm Then use 300–500 μm drug-loaded microspheres. Drug-loaded microspheres are divided into domestic Calli Spheres drug-loaded microspheres and imported DCB drug-loaded microspheres. In the cTACE group, epirubicin or raltitrexed emulsion mixed with lipiodol was injected under fluoroscopy monitoring for embolization. In addition to receiving interventional therapy for intervention, patients can take targeted therapy drugs as needed. If tumor progression is found, targeted drugs need to be replaced for second-line targeted drugs or immunotherapy.

### Survival analysis

The primary endpoints in the survival analysis were PFS and OS. During the follow-up period, PD and death were observed as the outcomes of PFS and OS, respectively, and the outcomes of patients lost to follow-up were listed as censored. The time of outcome events in the two groups was counted. In the DEB-TACE group, 16 patients had disease progression and 10 died. The longest survival time was 26.23 months and the shortest was 2.67 months. In the cTACE group, a total of 26 patients had tumor progression and 16 patients died, of which the longest survival time was 28.47 months and the shortest was 4.27 months.

**Figure 1:**
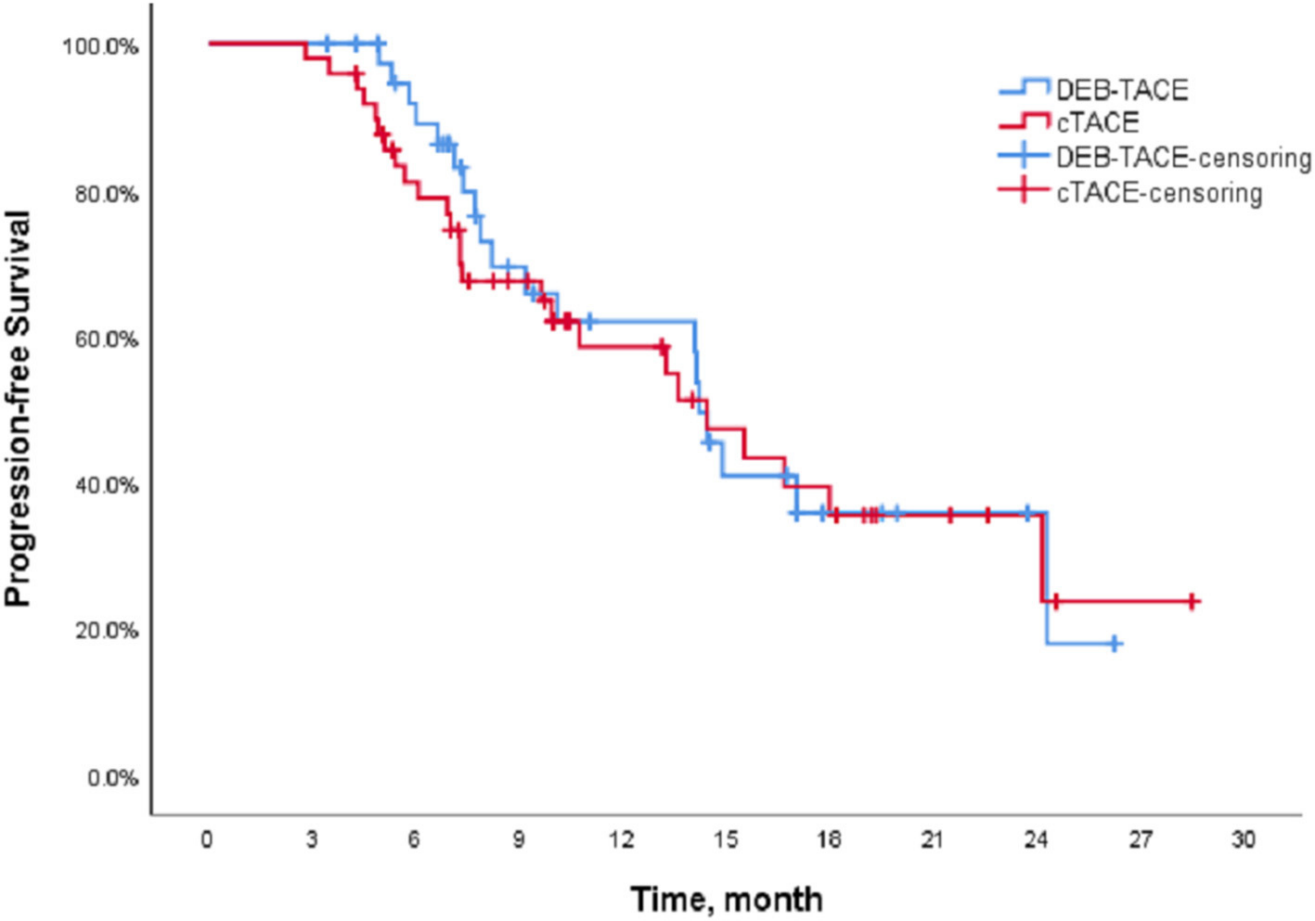
Progress free survival of the DEB-TACE and cTACE groups.

**Figure 2:**
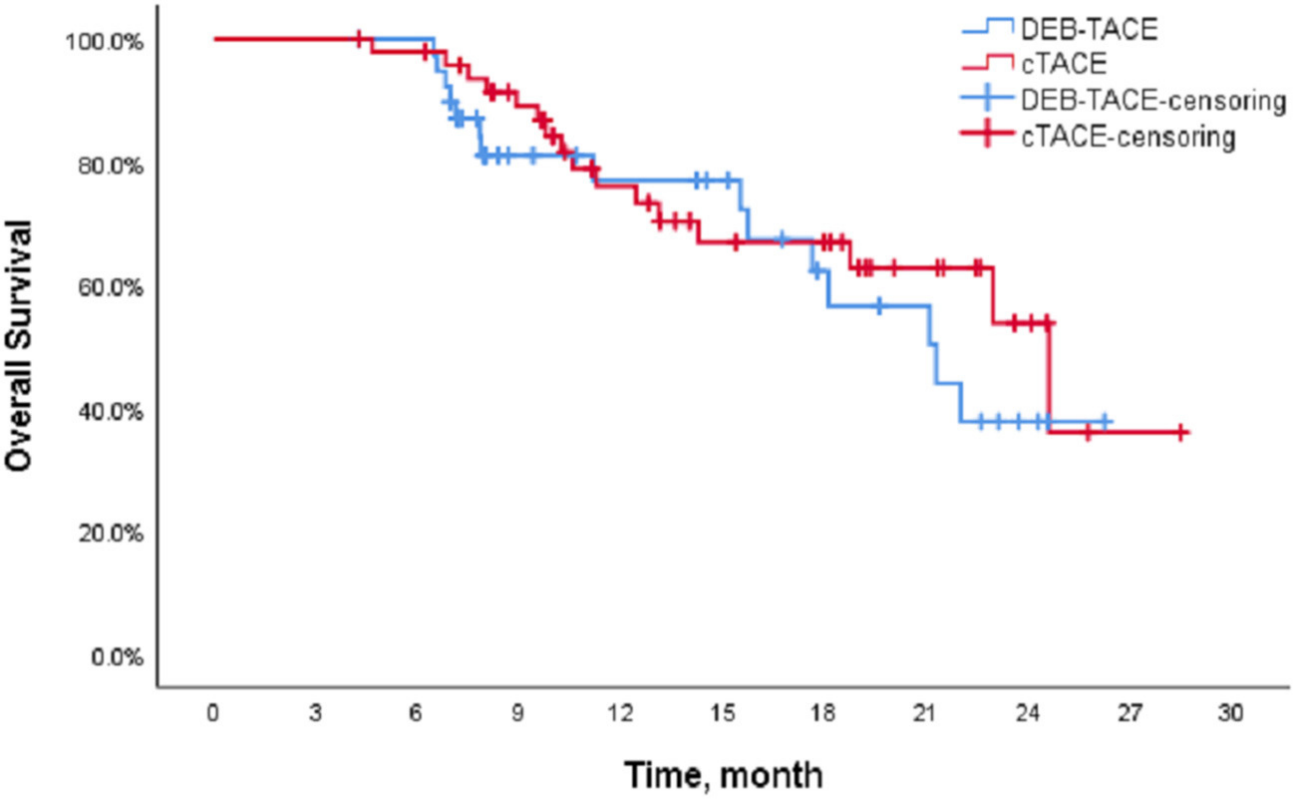
Overall survival of the DEB-TACE and cTACE groups.

The Kaplan-Meier (K-M) method was used to perform survival analysis of the outcome and event schedules of patients in the DEB-TACE and cTACE groups (Supplementary Tables 1, 2) using SPSS. According to the calculation, the median PFS of patients in the DEB-TACE group was 14.20 months (95% CI 13.316–15.084), and it was 14.43 months (95% CI 9.162–19.698) in the cTACE group. There was no significant difference in disease progression (*P* = 0.728). In addition, the mean survival time of DEB-TACE group and cTACE group were 19.18 ± 1.34 and 20.82 ± 1.42 months, respectively, and the median OS was 21.27 months (95% CI 15.718–26.822) and 24.6 months (95%), respectively, CI 17.607–31.593), and the Log-Rank test showed that there was no significant difference in the overall survival rate between the two groups (*P* = 0.411). The K-M curves of PFS and OS of the two groups of patients are shown in Figures 1, 2, respectively.

### Model structure

**Figure 3:**
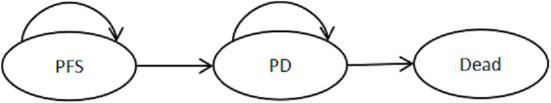
Partition survival state transition model.

Partition survival models (PSM) belong to the category of Markov models and are often used for economic evaluation of tumors. Compared with the Markov model, the PSM does not require a hypothetical estimate of the transition probability from one healthy state to the next, but by partitioning the raw survival data or the progression-free survival curve and the overall survival curve, Obtaining the specific number or proportion of individuals in each health state avoids the influence of model assumptions on research results (14). Therefore, the PSM is used for cost-effectiveness analysis. The PSM is usually divided into three health states: PFS, disease progression (PD), and Death, as shown in Figure 3 (15). Based on real-world clinical data, this paper can directly obtain the number of patients with HCC in the three health states of PFS, PD and death, and then obtain the corresponding health output and cost. Assuming that all patients were in the PFS stage at the start of the study, the final status of all patients was death. Building a partition survival model for cost-effectiveness analysis is to make decisions based on the results of incremental analysis, mainly calculating the Incremental Cost Effectiveness Ratio (ICER), using ICER to represent the cost of each additional quality-adjusted life years (QALY) (16). Calculated as follows:


(1) $$\begin{array}{r} ICER=\frac{C_{1}-C_{2}}{E_{1}-E_{2}} \end{array}$$

Through follow-up, the disease progression and survival of patients during the follow-up period can be known. In order to simulate the entire life cycle of patients, it is necessary to fit the survival of patients. The individual patient data were analyzed using the surveyHE data package in the R language, and the Log-normal parameter distribution was obtained as the best fitting model (17). The parameters of the log-normal parametric distribution were calculated to yield the meanlog (μ) and sdlog (σ) (18) (Table 2). Then, the μ and σ values calculated by the two groups of PFS and OS were substituted into the survival function of the Log-normal parameter distribution for fitting calculation. Calculations found that when the simulation time was 10 years, the mortality rate of patients in both groups exceeded 98%, so the study time was set to 10 years. The partition survival model (PSM) was constructed using Microsoft EXCEL.

**Table 2:** Parameter values of Log-normal parameter distribution.

**Group**	**PFS**		**OS**	
	**μ**	**σ**	**μ**	**σ**
DEB-TACE	2.741576	0.786687	3.227158	0.742339
cTACE	2.59371	0.804045	3.11888	0.719892

### Model parameters

#### Cost

Costs were collected by going to the Interventional Radiology Department of Cancer Hospital Affiliated to the University of Chinese Academy of Sciences. The costs of treatment during the follow-up period of the 89 patients included were collected one by one. The required direct medical costs include registration fees, diagnosis and treatment fees, inspection fees, hospitalization fees, interventional surgery fees (DEB-TACE and cTACE and other treatment methods), drug fees and other costs. After interventional surgery, daily liver protection drugs and anticancer drugs need to be taken orally; if they have HBV, they need to take anti-HBV drugs continuously; Sorafenib and lenvatinib are mainly used for first-line treatment, and regorafenib and tislelizumab are used for second-line treatment, all of which belong to drug costs. In addition to this, the management costs of adverse reactions of grade 3 to 4 after treatment need to be considered, as shown in Table 3.

**Table 3:** The main unit cost.

**Medical project**	**Unit cost/$**
Admission check/follow-up check	162.11
Basic hospital expenses	25.8
**Interventional treatment costs**	
DEB-TACE	5592.4
cTACE	2624.4
Post-operative maintenance costs	75.97
**Drug cost**	
Bicyclool	9.59
Ganfule capsule	14.62
Compound Glycyrrhizin Tablets	4.17
Ci Dan capsule	21.3
Tenofovir Disoproxil Fumarate Tablets	1.7
Sorafenib	204.19
Renvatinib	483.26
Regorafenib	720.33
Tislelizumab	325.16
**Adverse event management costs**	
Morphine Hydrochloride Injection	0.53
Metoclopramide hydrochloride injection	0.35
Ondansetron Hydrochloride Tablets	15.8
Indomethacin suppository	0.94
Lactulose Oral Solution	4.77
Nitroglycerin Sublingual Tablets	0.59

There are certain differences in the frequency or dosage of interventional therapy and the use of targeted drugs and/or immunotherapy for each patient. The cost of treatment and medication and the management cost of adverse reactions were integrated for the two groups of patients with PFS and PD, respectively. These adverse reactions were alleviated by drug treatment, therefore, this cost was only considered in the first cycle in the partitioned survival model.

However, the sample size of the two groups of patients is small. If the cost is directly calculated to take the mean or median, the applicability of the cost cannot be objectively reflected. Therefore, the bootstrap method was used here to calculate the total cost of 1,000 samples consumed by patients in PFS and PD health status, and the corresponding cost mean and 95% confidence interval were obtained (19). The sampling results show that the PFS cost of a single cycle DEB-TACE group is 599.97 $, the PD cost is 162.75 $, and the AEs management cost is 3.28 $. The PFS cost, PD cost and AEs management cost of cTACE group were 353.88, 247.64 and 3.84 $, respectively.

#### Utility

In this study, the EQ-5D-5L questionnaire was distributed to investigate the health utility value of patients in PFS and PD health status and treated with DEB-TACE or cTACE. A total of 152 questionnaires were collected (20). Among them, the DEB-TACE group had 35 PFS health status and 29 PD health status; the cTACE group had 56 and 32 PD health status, respectively. During the investigation, the negative effect of postoperative adverse reactions has been reflected in the questionnaire results, so it will not be considered again. Use the health utility score system suitable for the Chinese population studied by Luo et al. to calculate the utility value, and then use bootstrap to sample 4 groups of samples 1,000 times (21). Similarly, for small sample sizes, bootstrap is used to sample 1,000 samples from 4 groups. The mean utility values for PFS and PD in the final output DEB-TACE group were 0.8773 (95% CI: 0.8410, 0.9109) and 0.8228 (95% CI: 0.7902, 0.8536), respectively. The mean utility values for PFS and PD health status in the cTACE group were 0.8123 (95% CI: 0.7911–0.8345) and 0.7898 (95% CI: 0.7560–0.8197), respectively.

#### Discount

In order to compare and analyze the cost and health output at the same time node, according to the suggestion on the value of the discount rate in the evaluation of pharmacoeconomics in my country, the cost and health output will be discounted at an annual discount rate of 5.2% from the second year (22). All parameter values are shown in Table 4.

**Table 4:** Summary of costs and utility values.

**Parameter**	**Value**	**Lower**	**Upper**	**Distribution**
C_DEB-TACE_PFS	6339.96	5071.96	7607.95	Gamma
C_DEB-TACE_PD	3154.42	2523.54	3785.31	Gamma
C_cTACE_PFS	5528.42	4422.74	6634.1	Gamma
C_cTACE_PD	2274.2	1819.36	2729.04	Gamma
C_DEB-TACE_AE	3.28	2.62	3.93	Gamma
C_cTACE_AE	3.84	3.07	4.61	Gamma
U_DEB-TACE_PFS	0.1271	0.1144	0.1399	Beta
U_DEB-TACE_PD	0.1192	0.1073	0.1312	Beta
U_cTACE_PFS	0.1177	0.106	0.1295	Beta
U_cTACE_PD	0.1145	0.103	0.1259	Beta
U_Dead	0	0	0	Beta
Discount	0.75%	0.30%	1.20%	

### Sensitivity analysis

Considering the uncertainty of medical cost, we assumed the upper and lower bounds of medical cost to be ±20%. According to literature reports, the discount rate should be in the range of 2.1% to 8.3% for sensitivity analysis (22). One-way sensitivity analysis and probabilistic sensitivity analysis were used to explore the influence of each parameter on the model. Substitute the upper and lower limits of each parameter into the model for One-way sensitivity analysis and calculation; Probabilistic sensitivity analyses were performed using Monte Carlo simulations (*N* = 1,000 iterations) to analyze which drugs had a cost-effectiveness advantage at a willingness-to-pay (WTP) threshold. And the cost-effectiveness acceptability curve was used to estimate the optimal treatment measures in different WTP ranges. According to the recommendation of the “*China Pharmacoeconomic Evaluation Guidelines (2020)”*, the ICER value is compared with the per capita gross domestic product (GDP) three times that of my country in 2020. Statistics from the National Bureau of Statistics show that my country's per capita GDP in 2020 will be 10,499.74 $. Therefore, WTP is set to 31499.23 $/QALYs.

## Result

### Cost-effectiveness analysis

The partition survival model was simulated for 10 years. The results are shown in Table 5. The cumulative cost of the DEB-ATCE group was 94,901.92 $, and the cumulative effect was 14.1032 QALYs; the cumulative cost and cumulative effect of the cTACE group were 73,823.61 $ and 12.4058 QALYs, respectively. Compared with the cTACE group, the incremental cost of the DEB-TACE group was 43,792.46 $, and the incremental effect was 1.6975 QALYs. The ICER was calculated to be 11,875.62 $/QALYs, which was lower than the WTP threshold (31,499.23 $/QALYs), indicating that DEB-TACE treatment of HCC patients is economical.

**Table 5:** Result of cost-effectiveness analysis.

**Group**	**Cost/**	**Utility/**	**Incremental**	**Incremental**	**ICER**
	**$**	**QALY**	**cost/$**	**Utility/QALY**	
DEB-TACE	94901.92	14.1032			
cTACE	73823.61	12.4058	43792.46	1.6975	11875.62

### Sensitivity analysis

#### One-way sensitivity analysis

**Figure 4:**
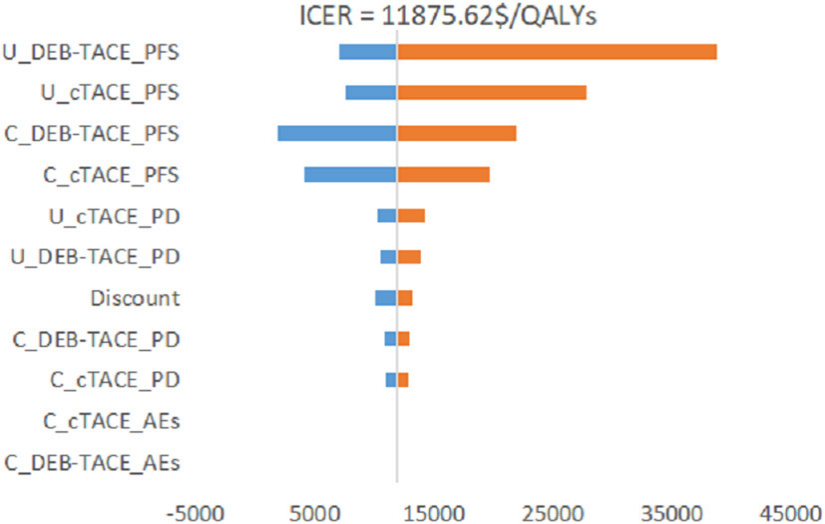
Tornado chart of one-way sensitivity analysis.

It can be seen from Figure 4 that the PFS utility value of the DEB-TACE group and the cTACE group is the biggest factor affecting the stability of the model. The cost changes of the other two groups of PFS states also have a certain impact, and the changes of other parameters have little effect. Among them, the change of the PFS utility value of the DEB-TACE group makes the output EV maximum value of 38678.29 $/QALY, which is greater than the WTP threshold (31499.23 $/QALYs).

#### Probabilistic sensitivity analysis

**Figure 5:**
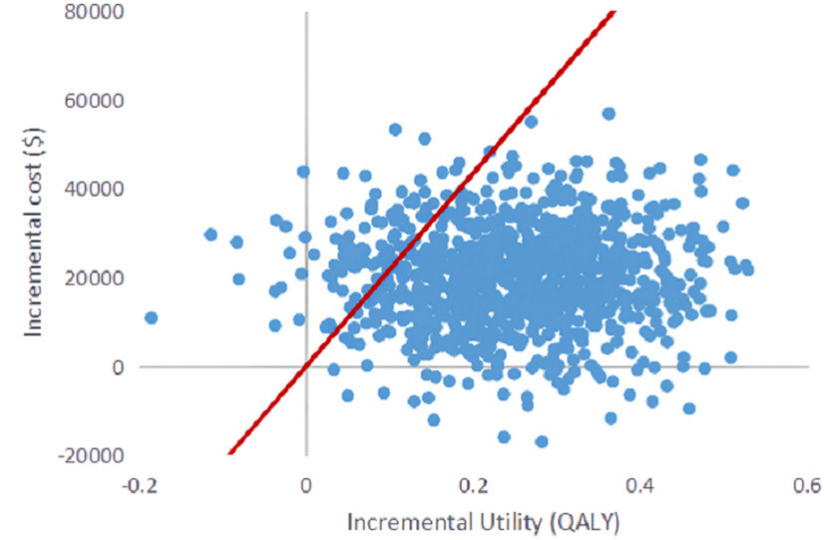
Scatter plot of incremental cost-effectiveness.

**Figure 6:**
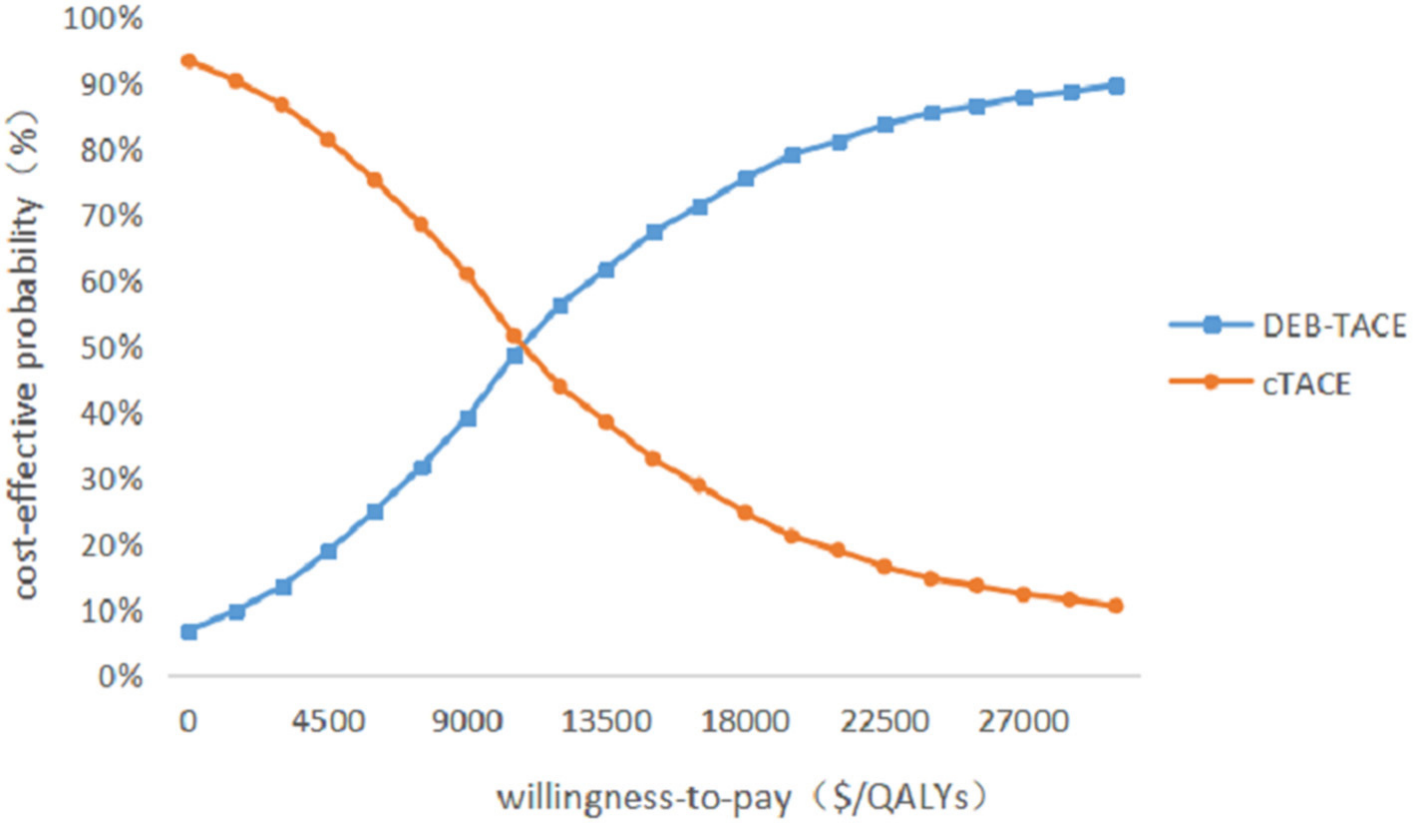
Cost-effectiveness acceptability curve.

Probabilistic sensitivity analysis was performed using 1,000 iterative Monte Carlo simulations, and Monte Carlo scatter plots and cost-effectiveness acceptability curves were drawn. It can be seen from Figure 5 that the incremental cost-effect scatter points are distributed on both sides of the WTP threshold. Comparing it with the WTP threshold, 92% of the incremental cost-effect scatter points are located on the lower right side of the WTP threshold. That is to say, the probability of DEB-TACE treatment of HCC patients is more cost-effective than 92%. In addition, the probability of the cost-effectiveness of the cTACE group gradually decreased with the increase of the WTP threshold. When the WTP value was <32,609.25 $/QALY, the cTACE group was more cost-effective than the DEB-TACE group. With the increase of the WTP threshold, the DEB-TACE group has an increasing probability of cost-effectiveness. When the WTP value is >65,218.50 $/QALY, the DEB-TACE group has a cost-effective probability of close to 90% (Figure 6).

## Discussion

The results of basic cases show that the BCLC staging of patients in the DEB-TACE group and the cTACE group is significantly different, and doctors usually recommend patients in stage A or B to choose cTACE for treatment. However, because the lipiodol treated by cTACE is liquid, it cannot completely block the blood flow, and the lipiodol in the tumor will gradually decrease with the blood flow, which cannot achieve the best therapeutic effect. The drug-loaded microspheres in DEB-TACE can be injected into the tumor feeding artery through the catheter to achieve sustained release of chemotherapeutic drugs, and permanently embolize the hepatic artery to obtain a higher tumor response rate (23). Therefore, when the patient's tumor condition is poor and the BCLC stage is B or C, DEB-TACE treatment is preferentially recommended. This may be the reason why the DEB-TACE group has no advantage in median PFS and median OS.

The cumulative cost and cumulative utility of the DEB-TACE group were greater than those of the cTACE group, with the cumulative cost of the two groups being 94,901.92 and 73,823.61 $, respectively; the cumulative utility was 14.1032 QALYs and 12.4058 QALYs, respectively. From this, it can be concluded that the incremental cost is 43,792.46 $, and the incremental effect is 1.6975 QALYs. Through the cost-effectiveness analysis method, the ICER value can be obtained to be 11,875.62 $/QALYs. In this paper, the willingness to pay threshold is set to be three times the per capita GDP of my country, that is, WTP is 31,499.23 $/QALYs. Comparing the ICER value with the WTP threshold, ICER < WTP indicates that the DEB-TACE group is economical.

Cucchetti A et al. (13) included 5 randomized controlled trials and 11 observational studies with a total of 1,860 patients with hepatocellular carcinoma and constructed a Markov model to assess the cost and efficacy of cTACE and DEB-TACE from a healthcare provider's perspective. The study results showed that the total cost of cTACE treatment was 10,389 euros, and the effect was 3.3 QALY; the total cost of DEB-TACE treatment was 11,418 euros, and the effect was 4.0 QALY. DEB-TACE is more cost-effective than cTACE when around 2,000–3,500 EUR/QALY is the minimum willingness to pay. This result is similar to that of our study, but ICER was not calculated and a sensitivity analysis was missing.

Since there are uncertainties in the methodology, cost, utility value, and discount rate in the model, sensitivity analysis is required for these uncertainties. Through the One-way sensitivity analysis, it can be seen that the two factors that have the greatest impact on the model are the utility value of the PFS health status of the DEB-TACE group and the cTACE group, followed by the cost of the PFS health status stage of the two groups. It can be seen from the incremental effects of the two groups that the DEB-TACE group has no obvious advantage in the utility value of the PFS health state. When the utility value is at the lowest value within the fluctuation range, the ICER value increases to 38,678.29 $/QALYs is greater than the WTP threshold, That is to say, it is not economical to perform DEB-TACE intervention if the patient is in a healthy state of PFS without good health. In addition, the ICER of the cTACE group PFS health status and the cost of the two groups of PFS health statuses within the set value range are smaller than WTP, which will not affect the stability of the model. In addition, using probability sensitivity analysis to sample the uncertainty parameters for 1,000 iterations of Monte Carlo simulation, and output the incremental cost-effect scatterplot, we can see that when the WTP is 31,499.23 $/QALYs, DEB-TACE is effective in the treatment of HCC. The probability of being economical is 92%. According to the cost-effectiveness acceptable curve, when the WTP is <32,609.25 $/QALYs, the cTACE group is more cost-effective than the DEB-TACE group; when the WTP is >32,609.25 $/QALYs, the DEB-TACE group increases with the WTP. The probability of being cost-effective gradually approaches 90%. Therefore, DEB-TACE is more economical while ensuring the health of patients.

The limitations of this paper have the following three points. First, on the screening of clinical patients. In this paper, the cases of real-world patients are collected as data, but retrospective screening will have a certain bias, and patients may have incomplete case reports during the real treatment process, which will have a certain impact on the results. Therefore, bias needs to be reduced by expanding the sample size. Second, on the fitting of survival data. In this paper, in order to simulate the 10-year survival of patients, the actual progression-free survival and overall survival of the patients were analyzed by parametric method, and the survival data were fitted according to the optimal fitting parameter distribution model. There are some discrepancies in the data. Therefore, it is necessary to obtain specific survival data of patients through long-term follow-up. Third, about the measurement of utility value. In this paper, the EQ-5D-5L health scale is used to measure the health utility value of patients in the form of a questionnaire. However, due to insufficient sample size, bootstrap is used to perform 1,000 round-trip sampling to obtain the final value, which has a certain impact on the research results. Therefore, more scales need to be collected to be representative.

## Conclusion

In practical clinical applications, DEB-TACE is a treatment method that is preferentially recommended for patients with advanced hepatocellular carcinoma. Although the drug-loaded microspheres used in DEB-TACE are more expensive for embolization, the cost-effectiveness analysis can conclude that DEB-TACE is a more economical treatment option.

## Data availability statement

The original contributions presented in the study are included in the article/Supplementary material, further inquiries can be directed to the corresponding author/s.

## Ethics statement

Written informed consent was obtained from the individual(s) for the publication of any potentially identifiable images or data included in this article.

## Author contributions

GuolS provided resources and was in charge of project management. JW was in charge of data investigation and original text writing. XZ was in charge of data processing and validation. GuojS and ZD provided methodology, with GuojS overseeing the progress of the project. ZD was responsible for the final original review and editing. All authors contributed to the article and approved the submitted version.

## Funding

This work was supported by the National Natural Science Foundation of China (Grant No. 82072032).

## Conflict of interest

The authors declare that the research was conducted in the absence of any commercial or financial relationships that could be construed as a potential conflict of interest.

## Publishers note

All claims expressed in this article are solely those of the authors and do not necessarily represent those of their affiliated organizations, or those of the publisher, the editors and the reviewers. Any product that may be evaluated in this article, or claim that may be made by its manufacturer, is not guaranteed or endorsed by the publisher.
